# Fabrication of Self-Assembled BiFeO_3_/CeO_2_ Nanocatalytic Materials for Efficient Catalytic Dye Degradation

**DOI:** 10.3390/nano13182545

**Published:** 2023-09-12

**Authors:** Lin Li, Qi Li, Dongqing Wang, Yubo Zhang, Fei Li, Yaru Zhang, Tifeng Jiao

**Affiliations:** State Key Laboratory of Metastable Materials Science and Technology, Hebei Key Laboratory of Applied Chemistry, Hebei Key Laboratory of Nano-Biotechnology, Hebei Key Laboratory of Heavy Metal Deep-Remediation in Water and Resource Reuse, Yanshan University, Qinhuangdao 066004, China

**Keywords:** CeO_2_, BiFeO_3_, nanocatalytic materials, non-homogeneous Fenton catalysts, dye degradation

## Abstract

The catalytic treatment of wastewater serves as an effective way to solve the problem of water pollution, in which non-homogeneous Fenton catalysts are widely used. However, the activity enhancement of non-homogeneous Fenton catalysts still remains a great challenge. Herein, self-assembled BiFeO_3_/CeO_2_ nanocatalytic materials with different molar ratios were successfully fabricated by a suspension blending method, following which the structure evolution was determined by various characterizations. The catalytic degradation of methylene blue (MB), rhodamine B (RhB), and saffron T (ST) were performed over the BiFeO_3_/CeO_2_ nanocatalytic materials. It was found that the 0.2BiFeO_3_:0.8CeO_2_ nanocatalytic materials exhibited an 80.8% degradation efficiency for RhB. The 0.6BiFeO_3_:0.4CeO_2_ nanocatalytic materials reached 81.1% and 48.7% for ST and MB, respectively. The BiFeO_3_/CeO_2_ nanocatalytic materials also showed a good stability during several cycles. The combination of CeO_2_ with BiFeO_3_ led to an enhanced activity for dye degradation, probably due to the electron transfer from ≡Fe^2+^ to ≡Ce^4+^. This study provides a new approach to dye degradation by using Fenton catalytic systems.

## 1. Introduction

With rapid population growth, water consumption by industry, agriculture, and households continues to increase [1], leading to water pollution as a global issue [2]. Synthetic dyes are among the most harmful chemical pollutants in water [3], and water contaminated by them can cause serious health problems [4,5], hence the need for wastewater treatment. Methods of treating water pollutants can be divided into physical, chemical, and biological methods [6]. However, some organic pollutants are toxic and non-degradable, and cannot be treated by conventional physical or biological methods [7]. Still, advanced oxidation processes (AOPs) in chemical methods can solve this problem [8,9]. Studies have shown that the Fenton process is one of the most cost-effective AOPs [10]. However, the homogeneous Fenton reaction has some disadvantages, such as a narrow optimal pH range and the production of iron-containing sludge [11,12]. Therefore, research focused on heterogeneous Fenton catalysis [13]. The non-homogeneous Fenton reaction solves the problems of pH range extension and stability enhancement; so, it is more commonly used in wastewater treatment [14]. H_2_O_2_ can be activated by Fe(II) to form a Fenton reaction, and the resulting free radicals (e.g., ·OH^−^) are capable of efficiently degrading dye molecules, further advancing the development of wastewater treatment technologies [15]. Iron oxide, zeolite clay, immobilized iron, and carbon materials are widely used non-homogeneous Fenton catalysts, but many show weak catalytic activity [16]. Therefore, efficient catalysts must be prepared to treat dye wastewater [17].

As an important catalytic material, CeO_2_ has attracted the attention of researchers in various catalytic applications for its unique structure and redox properties [18,19,20]. Lin et al. prepared PdO/CeO_2_ catalysts using the deposition–precipitation method, tested them in the non-homogeneous Fenton degradation of acid orange 7 (AO7), and found that they could remove up to 50% of AO7 [21]. Zhu et al. prepared CuO/CeO_2_ catalysts by ultrasonic impregnation and found that the removal rate of diclofenac reached 86.62% by testing [22]. Li et al. prepared LaFeO_3_/3DOM CeO_2_ catalyst and found that it maintained effective catalytic degradation of MB after 10 cycles [23]. In addition, CeO_2_ of the same purity is cheaper than other oxides; so, CeO_2_ was chosen as the loading in this study [24,25]. BiFeO_3_ is one of the important perovskite-type oxides that can provide Fe^3 +^ irrespective of pH [26]. Therefore, many researchers have combined BiFeO_3_ with other nanomaterials for dye degradation [27]. Huang et al. successfully prepared CdSe QDs/BiFeO_3_ composite catalysts using the stirring impregnation method. The degradation of phenol by 0.5CBFO reached 98.5 as compared to 41.5% for BFO in 60 min [28]. Volnistem et al. synthesized a BiFeO_3_/Fe_3_O_4_ catalyst for degradation of methylene blue under visible light. The time required for the complete degradation of methylene blue solution was more than seven times faster for samples containing 20% Fe_3_O_4_ than that needed for BiFeO_3_ alone [29]. Therefore, CeO_2_ and BiFeO_3_ composite catalysts may achieve good results in dye degradation.

In this study, we fabricated BiFeO_3_/CeO_2_ nanocatalytic materials with different molar ratios for the catalytic degradation of MB, RhB, and ST. The BiFeO_3_/CeO_2_ nanocatalytic materials exhibited an enhanced activity, compared with BiFeO_3_ or CeO_2_ nanoparticles, which might benefit from the electron transfer from ≡Fe^2+^ to ≡Ce^4+^. This work offers an effective way for catalytic dye degradation over Fenton catalysts.

## 2. Experimental Section

### 2.1. Materials

Cerium(III) nitrate hexahydrate, bismuth(III) nitrate pentahydrate, ferric(III) nitrate nonahydrate, citric acid, MB, and ST were purchased from the Shanghai Aladdin Biochemical Technology Co., Ltd. (Shanghai, China). RhB, hydrogen peroxide, glycol, ammonium hydroxide (25 wt%), and methanol were purchased from the Sinopharm Chemical Reagent Co., Ltd. (Shanghai, China). Nitric acid and hydrochloric acid were purchased from the Tianjin Kermel Chemical Reagent Co., Ltd. (Tianjin, China). All chemicals were used as received without further purification.

### 2.2. Preparation of BiFeO_3_/CeO_2_ Nanocatalytic Materials

#### 2.2.1. Synthesis of CeO_2_ Nanoparticles

CeO_2_ nanoparticles were obtained using a homogeneous precipitation method. Typically, 10.91 g of Ce(NO)_3_·6H_2_O was dissolved in 100 mL of aqueous glycol (80 vol%) with vigorous stirring in a 50 °C water bath to obtain a transparent solution. Then, 25 mL of 3.0 M NH_3_·H_2_O was added dropwise into the above solution, followed by stirring for 24 h. The resulting precipitate was collected by repeatedly washing with water and centrifugation, followed by drying at 80 °C overnight. The dried precipitate was calcined in air at 500 °C for 1 h, following which the CeO_2_ nanoparticles were acquired.

#### 2.2.2. Synthesis of BiFeO_3_

BiFeO_3_ was prepared by a sol–gel method. In a typical synthesis, 3 mmol of Bi(NO)_3_·5H_2_O and 3 mmol of Fe(NO)_3_·9H_2_O were dissolved in 50 mL of 0.5 M HNO_3_ solution. Afterwards, it was gelatinized by adding 3 mmol of citric acid with vigorous stirring, following which the mixture was dried at 80 °C. The resulting powder was then calcined at 600 °C for 2 h to obtain BiFeO_3_.

#### 2.2.3. Synthesis of BiFeO_3_/CeO_2_ Nanocatalytic Materials

BiFeO_3_/CeO_2_ nanocatalytic materials with different molar ratios were acquired by a suspension blending method. BiFeO_3_ and CeO_2_ were typically put into separate methanol solutions for ultrasonication to obtain BiFeO_3_ particles and CeO_2_ nanoparticles, respectively. Then, the BiFeO_3_ and CeO_2_ suspensions with a designed molar ratio were mixed with vigorous stirring. The mixture was then dried at 80 °C through evaporation to obtain the BiFeO_3_/CeO_2_ nanocatalytic materials with a designed molar ratio (0.2:0.8, 0.4:0.6, 0.6:0.4, or 0.8:0.2), which was denoted as 0.2BiFeO_3_:0.8CeO_2_, 0.4BiFeO_3_:0.6CeO_2_, 0.6BiFeO_3_:0.4CeO_2_, or 0.8BiFeO_3_:0.2CeO_2_.

### 2.3. Characterization

X-ray diffraction (XRD) data were acquired using a Rigaku Smart Lab diffractometer with Cu K*α* radiation. Raman spectra were recorded with a dispersive Horiva Jobin Yvon LabRam HR800 microscope equipped with a He–Ne laser. Nitrogen physisorption was performed with a Micromeritics ASAP 2460 instrument at −196 °C. Scanning electron microscopy (SEM) images and the corresponding element mapping were performed with a field-emission Quanta FEG 250 microscope. Transmission electron microscopy (TEM) images were acquired using a Hitachi HT7700 microscope. X-ray photoelectron spectroscopy (XPS) data were obtained using a Thermofisher ESCALAB 250Xi instrument with monochromated Al K*α* radiation as the X-ray source.

### 2.4. Catalytic Tests for Dye Degradation

The catalytic effect of BiFeO_3_/CeO_2_ nanocatalytic materials was observed by catalyzing the dyes MB, RhB, and ST. Firstly, 20 mg of BiFeO_3_/CeO_2_ nanocatalytic materials with different molar ratios were weighed into 50 mL solutions with concentrations of RhB (12 mg L^−1^), MB (15 mg L^−1^), and ST (15 mg L^−1^), respectively. The mixture solutions were stirred until adsorption and desorption equilibrate in the dark. Subsequently, 30% H_2_O_2_ was added dropwise to the mixture to reach a concentration of 0.016 M, followed by a dropwise addition of diluted HCL (2.0 M); the pH of the experimental solution was adjusted around 3, and the centrifugal supernatant was isolated at the same intervals. Finally, the absorbance value at the maximum absorption wavelength of the dye was measured, and the removal rate of the dye was calculated and fitted to the kinetic data according to Equations (1)–(3). The catalytic rates of nanocatalytic materials for different dyes were calculated by Equation (1).

(1) $$K=\frac{\left(A_{0}-A_{T}\right)}{A_{0}}\times 100\%$$

where K represents the catalytic rate, A_0_ represents the absorbance concentration of the original dye solution, and A_T_ represents the absorbance at the moment T.

We investigated the adsorption properties of these nanomaterials on the dye solutions by a quasi-first-order kinetic model and a quasi-second-order kinetic model.

Equation (2) is a quasi-first-order kinetic equation:

(2) $$q_{t}=q_{e}(1-e^{-k_{1}t})$$

Equation (3) is a quasi-second-order kinetic equation:

(3) $$\frac{t}{q_{t}}=\frac{1}{k_{2}q_{e}^{2}}+\frac{t}{q_{e}}$$

where q_e_ is the adsorption capacity of MB and RhB dyes at equilibrium (mg/g), q_t_ is the adsorption capacity of MB and RhB dyes at time t (mg/g), and the values of k_1_ and k_2_ are the quasi-first and quasi-second-order kinetic rate constants, respectively.

When the catalytic process was finished, the cyclic stability experiments of the BiFeO_3_/CeO_2_ nanocatalytic materials were carried out. The composite catalyst with the best catalytic effect was selected, washed with ethanol, and then washed and dried using ultrapure water. The recovered nanocatalytic materials catalysts were then catalyzed for six cycles under the same conditions for the three dyes.

## 3. Results and Discussion

**Figure 1:**
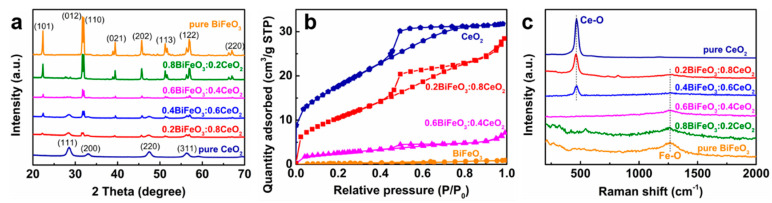
(**a**) XRD patterns of CeO_2_, BiFeO_3_, and BiFeO_3_/CeO_2_ nanocatalytic materials. (**b**) N_2_ adsorption–desorption isotherms for CeO_2_, BiFeO_3_, and BiFeO_3_/CeO_2_ nanocatalytic materials. (**c**) Raman spectra of CeO_2_, BiFeO_3_, and BiFeO_3_/CeO_2_ nanocatalytic materials.

Next, we performed various structural characterizations of the prepared CeO_2_ nanoparticles, BiFeO_3_ nanoparticles, and BiFeO_3_/CeO_2_ nanocatalytic materials. Figure 1a shows the XRD patterns of CeO_2_ nanoparticles, BiFeO_3_ nanoparticles, and four BiFeO_3_/CeO_2_ nanocatalytic materials with different molar ratios. There are three prominent absorption peaks of CeO_2_ with 2θ of 28.54°, 47.48°, and 56.33° corresponding to the crystallographic planes (111), (220), and (311) in the synthesized nanocatalytic materials. The XRD patterns of CeO_2_ nanoparticles do not have peaks corresponding to any secondary or impurity phases. The XRD patterns of BiFeO_3_ particles agree with the JCPDS data (Card No. 71-2494) and reveal a rhombic crystal structure of the BiFeO_3_ phase with the R3c space group. The well-defined XRD peaks of BiFeO_3_ particles indicate their enhanced crystallinity. The characteristic diffraction peaks of BiFeO_3_ and CeO_2_ crystalline forms can be represented by the XRD patterns of BiFeO_3_/CeO_2_ nanocatalytic materials. As shown in the figure, the diffraction pattern of the nanocatalytic materials is very similar to that of pure CeO_2_ at a BiFeO_3_ molar ratio of 0.2. When increasing the molar ratio of BiFeO_3_ in the nanocatalytic material samples, the diffraction patterns are more similar to those of pure BiFeO_3_, and the peaks at the crystalline plane at (101), (012), (110), and (104) are gradually enhanced. This may be due to the high crystallinity of the BiFeO_3_ phase and therefore shows as the dominant peak in the XRD spectra of the samples of the nanocatalytic materials.

Figure 1b shows the N_2_ adsorption–desorption isotherms of CeO_2_ nanoparticles, BiFeO_3_ nanoparticles, and BiFeO_3_/CeO_2_ nanocatalytic materials, and the test results are shown in Table 1. As displayed in Figure 1b, the adsorption–desorption of N_2_ occurred for CeO_2_ nanoparticles and BiFeO_3_/CeO_2_ nanocatalytic materials, but no adsorption–desorption of N_2_ occurred for BiFeO_3_ particles. The adsorption and desorption curves overlap at the relative pressure P/P_0_ < 0.46, mainly due to the reversible monomolecular layer adsorption. As seen in the figure, 0.2BiFeO_3_:0.8CeO_2_ adsorbs more N_2_ than 0.6BiFeO_3_:0.4CeO_2_ and the curve of the BiFeO_3_/CeO_2_ nanocatalytic materials has a significant hysteresis at higher relative pressures (P/P_0_ = 0.50–0.8), which implies that the nanocatalytic materials have a mesoporous structure. The CeO_2_ nanoparticle adsorption belongs to a type IV adsorption, but for the BiFeO_3_ nanoparticles, N_2_ adsorption and desorption were not significant. This may be due to the smooth, flaky nature of BiFeO_3_ nanoparticles.

Figure 1c shows the Raman spectra of CeO_2_, BiFeO_3_, and four different molar ratios of BiFeO_3_/CeO_2_ nanocatalytic materials. As shown in the figure, the vibration of the Ce–O bond gradually increases, and the vibration of the Fe–O bond gradually decreases with the increase in the BiFeO_3_ ratio. The successful preparation of the nanocatalytic materials BiFeO_3_/CeO_2_ was further verified.

**Figure 2:**
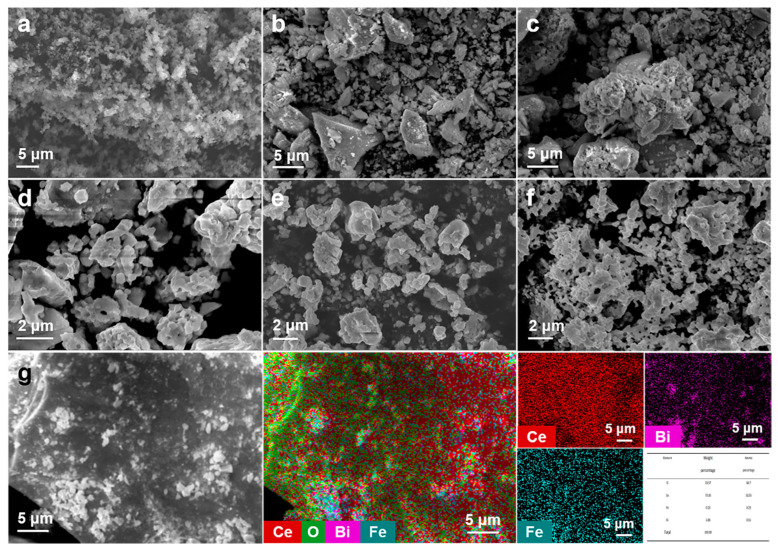
SEM images of (**a**) CeO_2_, (**b**) 0.2BiFeO_3_:0.8CeO_2_, (**c**) 0.4BiFeO_3_:0.6CeO_2_, (**d**) 0.6BiFeO_3_:0.4CeO_2_, (**e**) 0.8BiFeO_3_:0.2CeO_2_, and (**f**) BiFeO_3_. (**g**) SEM image of the 0.4BiFeO_3_:0.6CeO_2_ and the corresponding elemental content and mapping of Ce/Bi/Fe in catalytic material.

The SEM images of CeO_2_ nanoparticles, BiFeO_3_ nanoparticles, and BiFeO_3_/CeO_2_ nanocatalytic materials are shown in Figure 2. Figure 2a indicates that the CeO_2_ nanoparticles are spherical in shape, small in size, and clustered together. Figure 2b shows the SEM image of the 0.2BiFeO_3_:0.8CeO_2_ nanocatalytic materials, which has a large particle shape and attached CeO_2_ nanoparticles. Figure 2c shows the SEM image of 0.4BiFeO_3_:0.6CeO_2_ nanocatalytic materials, which shows the structure of pores between each large particle and the attachment of CeO_2_ nanoparticles. Figure 2d shows the SEM image of 0.6BiFeO_3_:0.4CeO_2_ nanocatalytic materials; it can be seen that this nanocatalytic materials appear with many pore structures and different lamellae agglomerated together with a small amount of CeO_2_ nanoparticles attached. Figure 2e shows the SEM image of the 0.8BiFeO_3_:0.2CeO_2_ nanocatalytic materials, where it can be observed that the morphology is a structure of lamellae superimposed on each other and does not have holes. There is a significant difference in the morphology of BiFeO_3_/CeO_2_ nanocatalytic materials with different molar ratios, which may be related to the amount of CeO_2_ nanoparticles and the speed and time of stirring. Figure 2f shows the SEM images of the BiFeO_3_ particles prepared by the sol–gel method, and it is observed that the morphology is irregularly clustered together in sheets and has a pore-like structure. Figure 2g shows the mapping image of the BiFeO_3_/CeO_2_ nanocatalytic materials, and similar elemental distribution results were obtained from different positions in samples. And it can be concluded that the presence of Ce, Fe, and Bi elements proves the successful preparation of the nanocatalytic materials BiFeO_3_/CeO_2_.

**Figure 3:**
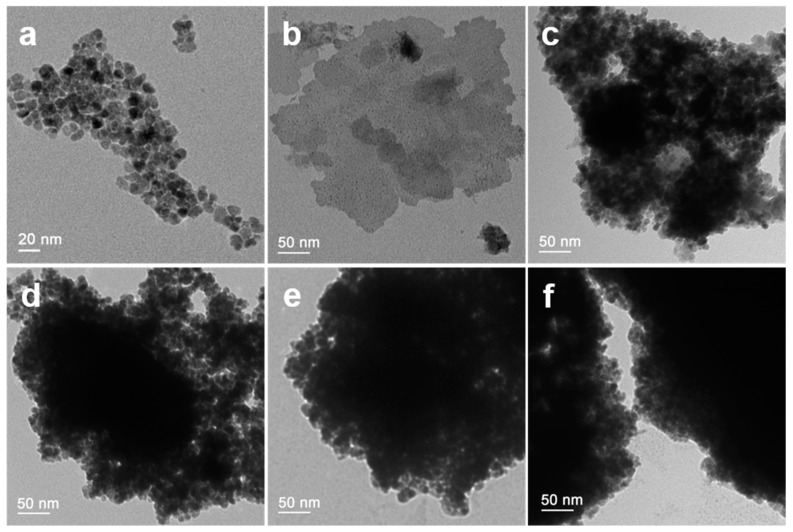
TEM images of (**a**) CeO_2_, (**b**) BiFeO_3_, (**c**) 0.2BiFeO_3_:0.8CeO_2_, (**d**) 0.4BiFeO_3_:0.6CeO_2_, (**e**) 0.6BiFeO_3_:0.4CeO_2_, and (**f**) 0.8BiFeO_3_:0.2CeO_2_.

The TEM images of CeO_2_ nanoparticles, BiFeO_3_ nanoparticles, and BiFeO_3_/CeO_2_ nanocatalytic materials are shown in Figure 3. Figure 3a shows the TEM image of CeO_2_ nanoparticles, which can be observed as CeO_2_ nanoparticles are spherical in shape with diameters ranging from 5–10 nm. Figure 3b shows the TEM image of BiFeO_3_ particles, which can be observed to be irregularly flaky and with a diameter of about 0.2 µm. Figure 3c–f show the TEM images of BiFeO_3_/CeO_2_ nanocatalytic materials with different scales, from which it can be observed that many CeO_2_ nanoparticles are attached to BiFeO_3_ particles, which, combined with the SEM images, further indicates that BiFeO_3_/CeO_2_ nanocatalytic materials are successfully prepared.

**Figure 4:**
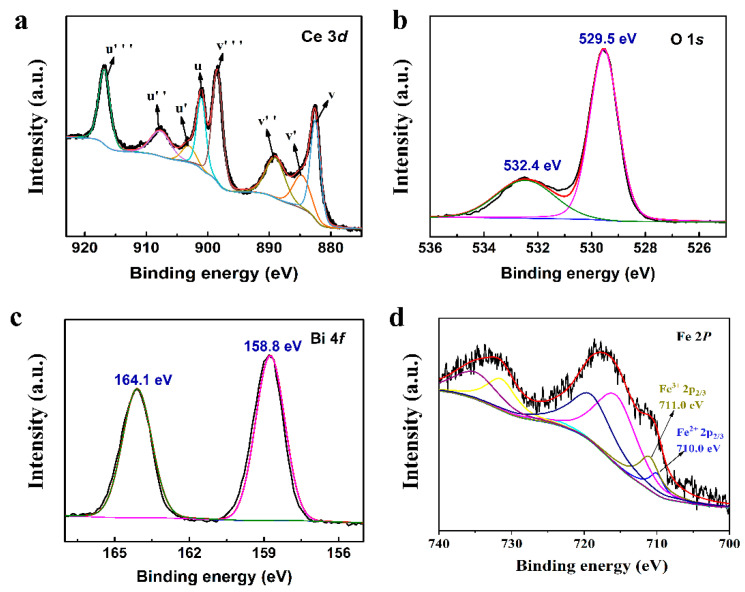
XPS of the 0.6BiFeO_3_:0.4CeO_2_ nanocatalytic materials. (**a**) Ce 3*d*. (**b**) O 1*s*. (**c**) Bi 4*f*. (**d**) Fe 2*p*.

Figure 4 shows the XPS spectrum of the 0.6BiFeO_3_:0.4CeO_2_ nanocatalytic materials. As shown in Figure 4a, the Ce 3d spectrum is decomposed into eight groups. The Ce 4f orbitals interact with the O 2p hybridization, and the four peaks labeled v (882.4 eV), v′ (885.3 eV), v″ (889.0 eV), and v‴ (898.4 eV) belong to Ce 3d_5/2_, and the peaks labeled u (900.7 eV), u′ (903.6 eV), u″ (907.3 eV), and u‴ (916.7 eV) are assigned to Ce 3d_3/2_. Of the eight peaks, v′ and u′ originate from the Ce^3+^ ion (Ce_2_O_3_), while the remaining six peaks correspond to the Ce^4+^ species (CeO_2_) [30]. The concentration of Ce^3+^ can be related to the surface oxygen vacancies; so, the formation of oxygen vacancies is always accompanied by the formation of Ce^3+^. In the BiFeO_3_ phase, the stable oxidation state of Fe is +3, which may also have Fe^2+^. This can also be confirmed by the Fe spectrum of its BiFeO_3_ nanostructure. The oxygen core spectrum in Figure 4b shows peaks corresponding to lattice oxygen (O_L_) and chemisorbed oxygen/other species (O_ads_) adsorbed on the surface of the BiFeO_3_/CeO_2_ nanostructure. Peaks of element O appear at 529.5 eV and 532.4 eV, indicating lattice (O_L_) and chemisorbed oxygen species (Oads), respectively. The XPS spectrum of Bi 4f of the sample is shown in Figure 4c. The characteristic spectrum of Bi4f consists of two characteristic peaks corresponding to the spin double Bi 4f_7/2_ and Bi 4f_5/2_, which correspond to binding energy magnitudes of 158.8 eV and 164.1 eV, indicating that the Bi element in the BiFeO_3_/CeO_2_ nanocatalytic materials is of +3 valence. As shown in Figure 4d, the peak of the Fe 2p_3/2_ line corresponding to Fe^2+^ appears at 710.0 eV, and the Fe 2p_3/2_ line corresponds to Fe^3+^ at 711.0 eV [31]. Figure 4d calculates Fe^3+^:Fe^2+^ = 55%:45%, which indicates that BFO has slightly more Fe^3+^ ions than Fe^2+^ particles. It indicates that the compounded BiFeO_3_ particles have multiple oxidation states (Fe^2+^/Fe^3+^), and Fe ions usually lead to oxygen vacancies in the BiFeO_3_.

**Figure 5:**
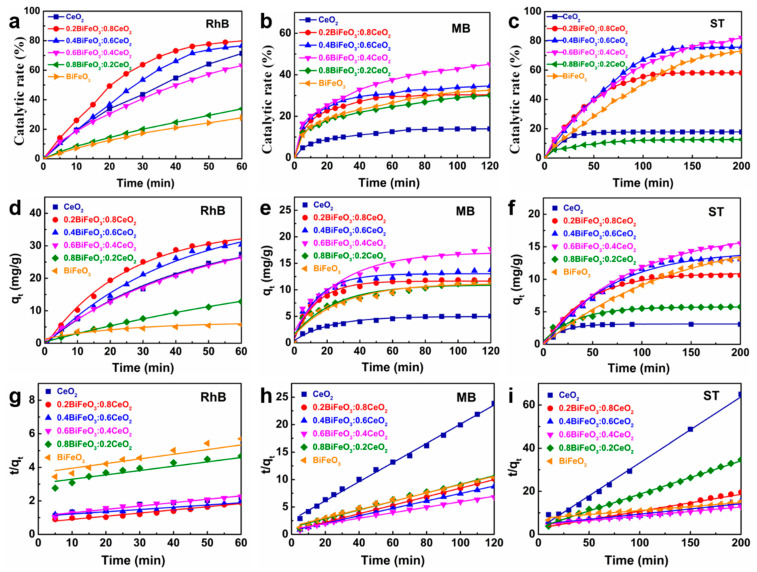
Adsorption kinetic results for degradation of (**a**,**d**,**g**) RhB, (**b**,**e**,**h**) MB, and (**c**,**f**,**i**) ST over CeO_2_, BiFeO_3_, and BiFeO_3_/CeO_2_ nanocatalytic materials.

The catalytic properties of CeO_2_ nanoparticles, BiFeO_3_ nanoparticles, and BiFeO_3_/CeO_2_ nanocatalytic materials on different dyes were investigated as follows. Figure 5 shows the adsorption kinetic results curves of CeO_2_, BiFeO_3_, and BiFeO_3_/CeO_2_ nanocatalytic materials for the degradation of RhB (a,d,g), MB (b,e,h) and ST (c,f,i). Table 2 shows its fitting according to the quasi-first-order kinetic Equation (2) and quasi-second-order kinetic Equation (3) to make the kinetic curves of each of the three dyes.

Figure 5a–c show that the catalytic rate of CeO_2_ nanoparticles, BiFeO_3_ particles, and BiFeO_3_/CeO_2_ nanocatalytic materials in catalyzing the dye solution started fast and then gradually slowed down to a stationary value. The fast catalytic rate at the beginning is because the concentration difference between the dye and the catalyst is relatively apparent, and the amount of H_2_O_2_ is sufficient to produce a large amount of ·OH by fully interacting with the catalyst under acidic conditions. As the catalytic reaction proceeds, the amount of H_2_O_2_ and the dye concentration gradually decrease; so, the catalytic rate slows. It can also be observed that there is a significant difference in the catalytic effect of CeO_2_, BiFeO_3,_ and different molar ratios of BiFeO_3_/CeO_2_ for other dyes. The catalytic efficiency of each molar ratio catalyst can only reach a maximum of 47.9% for MB dye solution within 120 min, while it can reach more than 80.0% for RhB and ST dye solutions. Among them, the nanocatalytic materials with a molar ratio of 0.2BiFeO_3_:0.8CeO_2_ could achieve the maximum catalytic efficiency of 80.8% for RhB within 60 min, while the nanocatalytic materials with a molar ratio of 0.6BiFeO_3_:0.4CeO_2_ could reach 81.1% and 48.7% for ST and MB dye degradation, respectively. As also shown in Figure 5, the correlation coefficients R^2^ of the fitted quasi-first-order and quasi-second-order models differed very little and were close to 1. Therefore, the quasi-first-order and quasi-second-order models could simulate the process of dye solutions catalyzed by CeO_2_, BiFeO_3,_ and BiFeO_3_/CeO_2_ nanocatalytic materials with different molar ratios more accurately. For RhB dye solutions, the catalytic amount of nanocatalytic materials with a molar ratio of 0.2BiFeO_3_:0.8CeO_2_ reaches 32.1 mg/g, larger than the other ratios. For MB and ST dye solutions, the nanocatalytic materials with a molar ratio of 0.6BiFeO_3_:0.4CeO_2_ had the highest catalytic amount per unit, reaching 17.1 mg/g and 17.4 mg/g, respectively. The effect of the catalysts with different molar ratios was different for other dyes, possibly due to the pH at the time of catalysis or the amount of H_2_O_2_ added.

**Figure 6:**
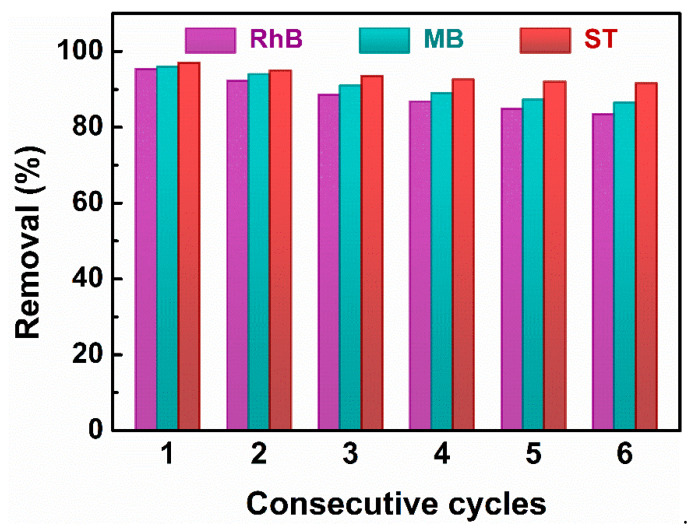
Cycling stability of the 0.6BiFeO_3_:0.4CeO_2_ nanocatalytic materials for degradation of RhB, MB, and ST.

The 0.6BiFeO_3_:0.4CeO_2_ catalysts at the end of the experiment were collected by centrifugation and then washed clean with anhydrous ethanol and ultrapure water before drying and collecting in an oven. The dried catalysts were repeated six times for the dye-catalyzed solution experiments under the same conditions, and the obtained experimental results are shown in Figure 6. The data show that after repeated cycling experiments conducted several times, the catalytic efficiencies of this sample for RhB, MB, and ST dye solutions were 83.5%, 88.7%, and 90.4% of the first time, respectively, indicating that the 0.6BiFeO_3_:0.4CeO_2_ nanocatalytic materials have good cyclable stability.

**Figure 7:**
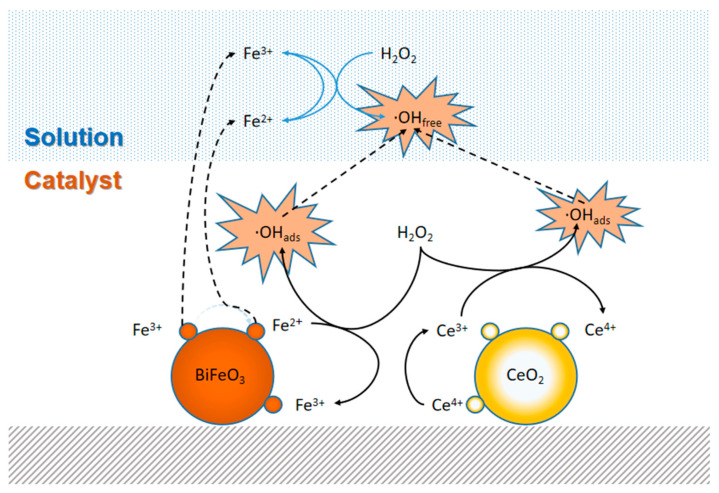
A proposed mechanism for catalytic dye degradation over the BiFeO_3_/CeO_2_ nanocatalytic materials.

Figure 7 shows the reaction mechanism of BiFeO_3_/CeO_2_ nanocatalytic materials for the activation of H_2_O_2_ under acidic conditions. First, the reaction of Fe^3+^ species with H_2_O_2_ (Equation (4)) and HO_2_·(Equation (5)) generates many ≡Fe^2+^ species. The standard redox potential of Ce^4+^/Ce^3+^ is 1.44 V, and Fe^3+^/Fe^2+^ is 0.77 V (Equations (6)–(8)). Therefore, the transfer of electrons from ≡Fe^2+^ to ≡Ce^4+^ (Equation (6)) is thermodynamically favorable [16]. Based on previous work, we know that cerium can cycle redox in the presence of H_2_O_2_ and produce ‧OH_ads_ (Equations (7)–(9)), which behaves similarly to iron in Fenton-like reactions [32]. The iron ions above BiFeO_3_ are dispersed in the native solution and trigger the decomposition of H_2_O_2_ by chain reactions (Equations (10)–(12)) to produce ‧OH free radical. A small number of ‧OH_ads_ can diffuse from the catalyst surface into the native solution. As shown in Equations (13)–(18), competing reactions may negatively affect oxidation. Finally, the hydroxyl radicals, which are self-carried on the catalyst surface and in the solution, decompose the dye solution.

≡Fe^3+^ + H_2_O_2_→ ≡Fe^2+^ + OH_2_·+ H^+^ (4)

≡Fe^3+^ + OH_2_·→ ≡Fe^2+^ + O_2_ + H^+^ (5)

≡Ce^4+^ + ≡Fe^2+^→ ≡Ce^3+^ + ≡Fe^3+^ (6)

≡Ce^3+^ + H_2_O_2_ + H^+^ → ≡Ce^4+^ +·OH_ads_ + H_2_O (7)

·OH_ads_ + H_2_O_2_ → HO_2_^−^ +H_2_O (8)

≡Ce^4+^ + HO_2_^−^→ ≡Ce^3+^ + H^+^ + O_2_ (9)

Fe^2+^ + H_2_O_2_ + H^+^→Fe^3+^ +·OH_free_ + H_2_O (10)

Fe^3+^ + H_2_O_2_→ Fe^2+^ + HO_2_·+ H^+^ (11)

Fe^3+^ + HO_2_·→ Fe^2+^ + O_2_ + H^+^ (12)

·OH + Fe^2+^→ OH^−^ + Fe^3+^ (13)

·OH + H_2_O_2_→ H_2_O + HO_2_· (14)

Fe^2+^ + HO_2_·→ Fe^3+^ + HOO^+^ (15)

HO_2_· + HO_2_·→H_2_O_2_^+^ + O (16)

·OH + HO_2_·(O_2_·^−^)→ O_2_ + H_2_O (+OH^−^) (17)

·OH +·OH→ H_2_O_2_ (18)

·OH + dye→ degraded products(19)

## 4. Conclusions

In conclusion, BiFeO_3_/CeO_2_ nanocatalytic materials with different molar ratios were synthesized and characterized using various techniques. The combination of CeO_2_ nanoparticles with BiFeO_3_ greatly increased the catalytic activity for the degradation of MB, RhB, and ST. The BiFeO_3_/CeO_2_ nanocatalytic materials also showed good stability during six consecutive cycles. The electron transfer from ≡Fe^2+^ to ≡Ce^4+^ within the BiFeO_3_/CeO_2_ nanocatalytic materials might dominate the catalytic activity of dye degradation. This finding provides an effective way for activity enhancement of catalytic degradation over Fenton catalytic systems.

## Figures and Tables

**Table 1:** Physisorption data of CeO_2_, BiFeO_3_, and BiFeO_3_/CeO_2_ nanocatalytic materials.

Sample	BET Surface Area (m^2^ g^−1^)	Total Pore Volume (cm^3^ g^−1^)	Average Pore Size (nm)
CeO_2_	65	0.049	3.03
0.2BiFeO_3_:0.8CeO_2_	40	0.044	4.44
0.6BiFeO_3_:0.4CeO_2_	9	0.011	4.68
BiFeO_3_	1	0.001	6.59

**Table 2:** Adsorption fitting data for degradation of RhB, MB, and ST over CeO_2_, BiFeO_3_, and BiFeO_3_/CeO_2_ nanocatalytic materials.

Dye	Catalyst	Pseudo-First-Order Kinetics			Pseudo-Second-Order Kinetics		
		q_e_ (mg g^−1^)	R^2^	K_1_ (10^−2^ min^−1^)	q_e_ (mg g^−1^)	R^2^	K_2_ (10^−4^ g mg^−1^ min^−1^)
RhB	CeO_2_	27.31	0.996	2.380	49.02	0.984	3.857
	0.2BiFeO_3_:0.8CeO_2_	32.13	0.995	4.048	52.94	0.958	5.050
	0.4BiFeO_3_:0.6CeO_2_	31.04	0.994	2.539	74.29	0.915	1.677
	0.6BiFeO_3_:0.4CeO_2_	26.29	0.999	2.087	49.95	0.988	3.661
	0.8BiFeO_3_:0.2CeO_2_	13.03	0.999	0.884	38.61	0.950	2.209
	BiFeO_3_	5.83	0.874	3.967	35.98	0.966	2.102
MB	CeO_2_	4.97	0.973	4.562	5.73	0.997	115.498
	0.2BiFeO_3_:0.8CeO_2_	11.65	0.978	7.274	12.85	0.999	92.706
	0.4BiFeO_3_:0.6CeO_2_	13.03	0.966	6.867	14.73	0.999	68.080
	0.6BiFeO_3_:0.4CeO_2_	17.09	0.958	3.557	19.93	0.992	26.842
	0.8BiFeO_3_:0.2CeO_2_	10.95	0.934	3.937	12.77	0.990	46.602
	BiFeO_3_	11.20	0.953	3.504	13.13	0.990	39.497
ST	CeO_2_	3.12	0.990	5.717	3.27	0.993	357.392
	0.2BiFeO_3_:0.8CeO_2_	10.96	0.997	2.288	12.71	0.976	21.482
	0.4BiFeO_3_:0.6CeO_2_	14.38	0.990	1.515	20.81	0.947	5.012
	0.6BiFeO_3_:0.4CeO_2_	17.44	0.998	1.114	23.98	0.988	3.969
	0.8BiFeO_3_:0.2CeO_2_	5.75	0.975	3.010	6.44	0.997	76.517
	BiFeO_3_	13.35	0.997	0.726	27.19	0.959	1.829

## Data Availability

Not applicable.
